# Brain response to taste in overweight children: A pilot feasibility study

**DOI:** 10.1371/journal.pone.0172604

**Published:** 2017-02-24

**Authors:** Cara Bohon

**Affiliations:** Department of Psychiatry and Behavioral Sciences, Stanford University School of Medicine, Stanford, California, United States of America; Peking University, CHINA

## Abstract

Understanding the neural response to food and food cues during early stages of weight gain in childhood may help us determine the drive processes involved in unhealthy eating behavior and risk for obesity. Healthy weight and overweight children ages 6–8 (N = 18; 10 with BMI between 5^th^ and 85^th^ %ile and 8 with BMI >85^th^ %ile) underwent fMRI scans while anticipating and receiving tastes of chocolate milkshake. Parents completed a Children’s Eating Behaviour Questionnaire. Results reveal greater response to milkshake taste receipt in overweight children in the right insula, operculum, precentral gyrus, and angular gyrus, and bilateral precuneus and posterior cingulate. No group differences were found for brain response to a visual food cue. Exploratory analyses revealed interactions between self-report measures of eating behavior and weight status on brain response to taste. This pilot study provides preliminary evidence of feasibility of studying young children’s taste processing and suggests a possible developmental shift in brain response to taste.

## Introduction

Obesity is associated with many negative health consequences and is resistant to treatment in adulthood [1], placing an importance on prevention interventions. Increased rates of pediatric obesity over the past 40 years [2] suggest that prevention must address early weight gain, but potential targets of prevention, the processes underlying unhealthy eating behaviors that lead to obesity in children, are not well understood. An improved understanding of these processes can help us develop prevention interventions to address them and improve eating behaviors and overall health in children.

Simply applying findings from adult literature to children is not appropriate, since the processes that maintain obesity in adults may not reflect those that occur early in the development of weight gain. For example, adults with obesity show hyper-response in reward regions to food cues, and a hypo-response to actual food intake [3], but lean adolescents at-risk for developing obesity by having two obese parents do not. Instead, they show greater activation to milkshake taste, compared to lean adolescents at lower-risk for developing obesity. No group differences were found in response to a cue signaling impending taste [4]. This may suggest that prior to the onset of obesity, individuals have hyper-response of reward circuitry to food taste, which diminishes after chronic overeating. Thus, obesity may develop when individuals experience strong pleasure from eating palatable foods, reinforcing motivation for eating. As overeating becomes more frequent, cues associated with food begin to stimulate reward response, as well, further increasing cravings. The response to cues that indicate subsequent food intake or the potential for food intake can be considered anticipatory reward. Over time, reward response to actual food receipt diminishes while anticipatory reward increases, driving overeating in attempt to experience the expected level of pleasure. This hypothesized developmental process is not fully understood, as few studies have measured neural response to food and food cues in children.

The studies that have examined brain response to food images or tastes in obese children found greater activation in prefrontal cortex to food images, which can be considered a food cue [5,6] and greater activation in the insula and amygdala in response to taste and in the frontal gyrus, paracingulate gyrus, and cuneus in response to sucrose taste in obese compared to lean children [7]. These three studies included children ages 10–17, 9–18, and 8–12 years respectively. Children of these age ranges may have engaged in unhealthy eating for a number of years, resulting in changes in brain response to food cues and taste. Indeed, a study of rates of pediatric obesity found that, of children who were obese by age 7, most were already obese by age 4[8]]. Thus, investigating neural response to food in even younger groups could reveal developmental processes. Additionally, studying neural processing in overweight children may provide insight into the processes that precede obesity.

The current study examines younger children (6–8 years old) to investigate early correlates of overweight (rather than obesity to examine early development of weight gain rather than consequences of obesity). The primary aims of the study were to (1) examine feasibility of studying taste processing at this age and (2) examine preliminary findings with implications for future research. We predicted that brain response to food cues (anticipatory reward) and tastes (consummatory reward) would be heightened in children with BMI > 85^th^ %ile compared to those in a healthy weight. Further, studies of neural response to food in children have not examined eating behaviors, which may be important in understanding neural activity in unhealthy weight gain. Thus, the current study explores the relation between Desire to Drink, Food Responsiveness, Enjoyment of Food, and Emotional Over-Eating subscales of the Child Eating Behaviour Questionniare (CEBQ) [9] and brain response to food cues and tastes in overweight and lean children.

## Materials and methods

The final sample consisted of 18 children ages 6–8 years (10 healthy weight BMI %ile range 5–73 [8 females]; 8 overweight BMI %ile range 88–95 [5 females]) recruited from the community. Recruitment initially included ages 4–5, but three children under age 6 years had to be excluded due to excessive head motion. The final sample comprised 5% Hispanic and 95% non-Hispanic; 5% Native Hawaiian/Pacific Islander, 17% mixed race (Asian/Caucasian), and 78% Caucasian. The sample as a whole came from relatively high socioeconomic status (SES) backgrounds, with 50% of the sample coming from a household making greater than $150,000 per year and only 17% from a household making less than $100,000 per year. The study was approved by the Stanford University Institutional Review Board. After a parent signed informed consent and children provided written assent (verbal assent if younger than age 7), children first went through a practice scan in a mock scanner to reduce motion in these young participants. Children watched a video while in the mock scanner, which was designed to feel like the real MRI scanner, and were alerted to head motion during the video via a bell. Once children were able to remain still for eight minutes (the duration of the longest scan), they competed a shortened version of the taste experiment in the mock scanner and practiced swallowing at the time noted on the screen, while keeping their head still. If they were not able to remain still for eight minutes, they did not participate in the functional magnetic resonance imaging (fMRI) scan. Six children were excluded after mock scan due to head motion or inability to follow safety instructions. Additionally, we had to exclude 13 children’s scan data from our final dataset due to excessive motion in the fMRI data. This included three children ages 4- and 5-years, resulting in the final dataset of 18 children ages 6–8 years. There were no differences in age, gender, or weight between children excluded and those in the final dataset.

Parents completed the Child Eating Behaviour Questionniare (CEBQ) [9], a 35-item measure describing eating behaviors related to obesity risk rated on a 5-point Likert scale ranging from Never to Always, while children underwent the mock scan procedure. Example items of the CEBQ include: My child loves food. My child eats more when worried. My child is always asking for food. My child is always asking for a drink. This scale provides subscale scores on Food Responsiveness, Emotional Over-Eating, Enjoyment of Food, Desire to Drink, Satiety Responsiveness, Slowness in Eating, Emotional Under-Eating, and Food Fussiness. Only the first four scales were used in this study, as they are more closely related to excessive eating and potential for weight gain. Further, behavioral measures of eating behavior were significantly related to scores on Food Responsiveness and Enjoyment of Food, suggesting that this measure is valid as a parent-report assessment of eating behaviors in children, especially for these subscales [9]. Parents also completed information about household income, used as a measure of socio-economic status (SES) and categorized into 5 levels.

In the fMRI procedure, identical to the paradigm used in prior studies [10, 11], participants were asked to consume regular meals, but refrain from eating or drinking caffeinated beverages for 4–6 hours immediately prior to their scan. This represents the hunger state that many individuals experience before their next meal. Two participants had eaten more recently and six had eaten more than 6 hours prior to scan. There were no group differences in hours since last eaten. Most participants were scanned between 16:30 and 18:30, but two overweight and four healthy weight participants were scanned between 11:00 and 15:00. The fMRI paradigms included stimuli presented in five runs. Participants viewed images of a glass of chocolate milkshake or a glass of water. Following 60% of the trials, corresponding tastes of 0.5cc of either a chocolate milkshake or a tasteless solution were delivered over 3 sec through a taste manifold. The chocolate milkshake was made of 1 cup vanilla Häagen Dazs ice cream, 1 cup 2% milk, and 2 tablespoons Hershey’s chocolate syrup. The tasteless solution was made of 0.45 gram Potassium Chloride and 0.10 gram of Sodium BiCarbonate into 500 mL of water. No taste followed the remainder of the images to allow the investigation of brain response to anticipatory reward that was not confounded with actual taste receipt. Tastes were delivered using two programmable syringe pumps to ensure consistent rate and timing of delivery through Tygon beverage tubing to a taste manifold attached to the head coil and placed in participants’ mouths. Visual images were presented for 3 sec followed by a 1–7 sec jitter, followed by a 5 sec delivery of the taste (or no taste) and a 1.5 sec presentation of instruction to swallow, and then another 1–7 sec jitter. Each run consisted of 20 trials total.

Scans were conducted on a 3-tesla GE scanner. Functional scans used a T2*-weighted gradient echo spiral pulse sequence [12] sensitive to BOLD contrast. Functional scans used the following parameters: TE = 30.0ms, TR = 2000ms, flip angle = 80°, FOV = 22.0cm, number of slices = 31, slice thickness = 4.0mm, acquisition matrix = 64x64. Structural scans used the following parameters: TE = minimum, TR = 8.1s, flip angle = 15°, FOV = 224cm, slice thickness = 1.6mm, 124 slices in the coronal plane, matrix = 256x192. Images were reconstructed as a 256 x 256 x 124 matrix. Shimming reduced blurring and signal loss from field inhomogeneities. Additionally, a high-resolution T1-weighted 3D inversion recovery spoiled gradient-recalled acquisition was collected as a structural image for registration purposes.

Analyses were performed using FEAT software (FMRI Expert Analysis Tool; FSL; www.fmrib.ox.ac.uk/fsl), and data were motion corrected, skull stripped, and spatially smoothed with a 5-mm full-width half-maximum Gaussian kernel. Image processing included mean-based intensity normalization and high-pass temporal filtering. Functional images were coregistered with structural images in native space and registered structural images to standard structural images (MNI-152). Contrasts included response to the image of the milkshake compared to water and the receipt of the milkshake taste compared to tasteless control. The swallow instruction was included as a regressor of non-interest to address potential head motion during swallow. All scan data underwent FSL’s MCFLIRT to correct for motion. If relative motion was greater than 1.5mm, we used FSL’s tool for removing motion outliers, using DVARS (RMS intensity from volume to volume, see [13]). If motion consisted of more than just these outliers (spikes), the data were not used.

Whole brain analyses were performed using mixed-effects modeling to account for inter-subject variability. Cluster-based correction/thresholding in FSL was used with a *p* < 0.05 and *z* > 1.7, due to the small sample and preliminary nature of the study. Contrasts between milkshake and water images and between milkshake and tasteless taste receipt were examined at the individual subject level before being combined for higher-level group analyses. Group comparisons were examined rather than using BMI as a continuous variable due to the bimodal distribution of BMI in the sample. Relation between CEBQ subscales and BOLD response to events was examined in FEAT at the group level by including the subscale scores as covariates of interest.

## Results

Chi-square tests of independence were performed to examine the relation between weight status and gender and weight status and socio-economic status (SES). Neither of these tests were significant (Χ^2^ (1, *N* = 18) = .678, *p* = .410 for gender; Χ^2^ (4, *N* = 18) = 2.925, *p* = .4570 for SES), suggesting no significant differences between weight groups on these variables.

**Fig 1:**
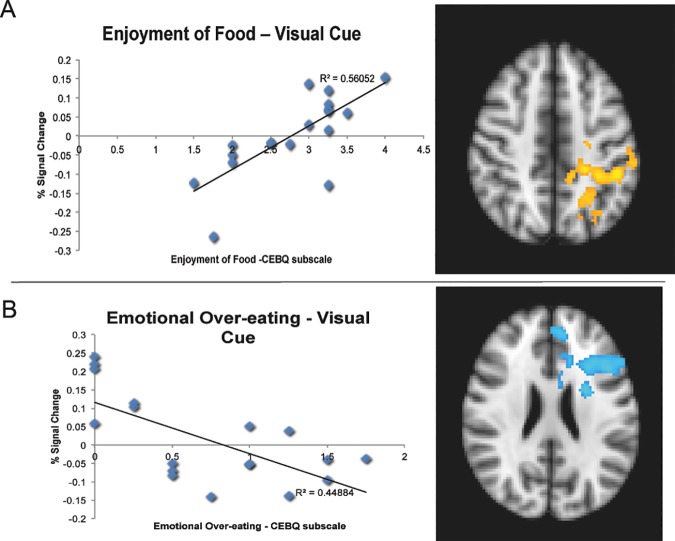
Relation Between CEBQ Scores and % Signal Change to Visual Cues Across Significant Clusters. Panel A depicts a scatterplot of the relation between scores on Enjoyment of Food subscale of the CEBQ and % signal change (average of the significant cluster from group-level FEAT results) in the left lateral occipital cortex, supramarginal gyrus, and insula/operculum (represented in yellow on brain image) in response to the milkshake picture vs water picture. Panel B depicts a scatterplot of the relation between scores on the Emotional Over-Eating subscale of the CEBQ and % signal change (average of the significant cluster from group-level FEAT results) in the left caudate/putamen, anterior cingulate cortex (ACC), middle frontal gyrus, and paracingulate gyrus (represented in blue on brain image) in response to the milkshake picture vs water picture. Brain images are in radiological format, where the left side of the brain is displayed on the right. All clusters are layered on to the MNI Standard brain for display purposes.

No significant group differences were found between healthy weight and overweight groups in response to images (milkshake picture compared to water picture). No relations were present between brain response to images (milkshake picture compared to water picture) and scores on Desire to Drink or Food Responsiveness. No interactions were present between weight status and any CEBQ subscale for response to images. There was a positive correlation across groups between Enjoyment of Food scores and brain response to milkshake > tasteless images in the left lateral occipital cortex, supramarginal gyrus, and insula/operculum (cluster size = 3492 voxels, *p* < .003, *Z* max = 2.71). There was a negative correlation across groups between Emotional Over-Eating scores and brain response to milkshake > tasteless images in the left caudate/putamen, ACC, middle frontal gyrus, and paracingulate gyrus (cluster size = 3029 voxels, *p* = .010, *Z* max = 2.78; Fig 1).

**Fig 2:**
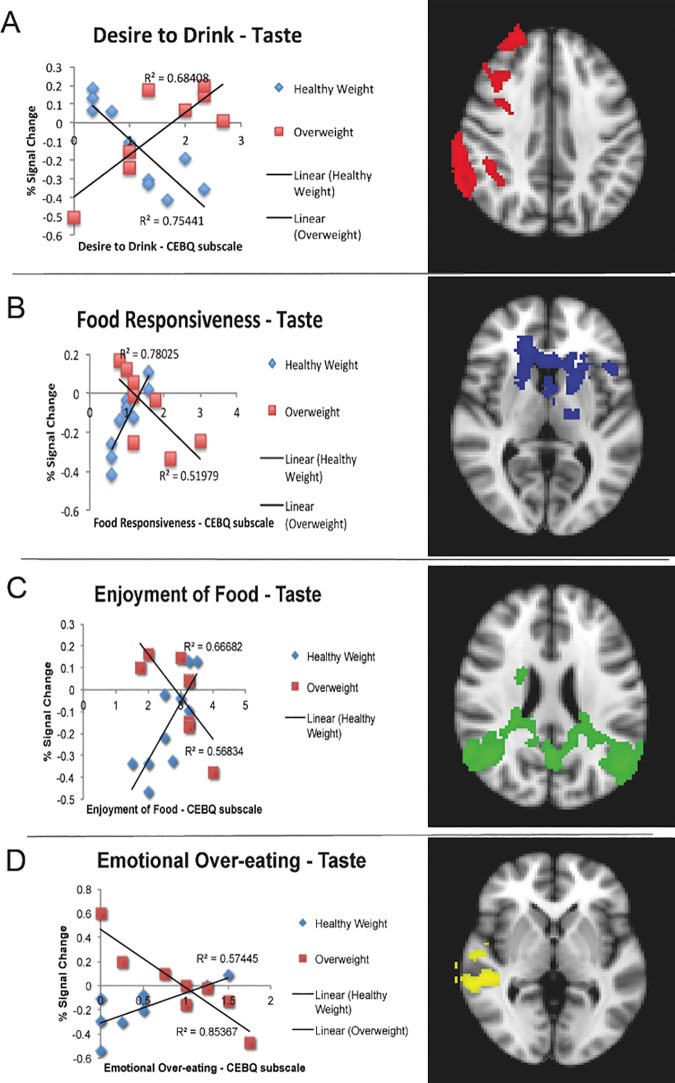
Relation Between CEBQ Scores and % Signal Change to Taste Receipt Across Significant Clusters. Panel A depicts a scatterplot of the interaction between scores on Desire to Drink subscale of the CEBQ and weight status and % signal change (average of the significant cluster from group-level FEAT results) in the right postcentral gyrus, parietal operculum, supramarginal gyrus, middle frontal gyrus, and frontal pole (represented in red on brain image) in response to receipt of milkshake taste vs tasteless receipt. Panel B depicts a scatterplot of the interaction between scores on the Food Responsiveness subscale of the CEBQ and weight status and % signal change (average of the significant cluster from group-level FEAT results) in the left putamen, frontal operculum, and postcentral gyrus, and the bilateral caudate and thalamus (represented in blue on brain image) in response to receipt of milkshake taste vs tasteless receipt. Panel C depicts a scatterplot of the interaction between scores on the Enjoyment of Food subscale of the CEBQ and weight status and % signal change (average of the significant cluster) in the bilateral occipital cortex, posterior cingulate, precuneus, angular gyrus, and right middle temporal gyrus (represented in green on brain image) in response to receipt of milkshake taste vs tasteless receipt. Panel D depicts a scatterplot of the interaction between scores on the Emotional Over-Eating subscale of the CEBQ and weigth status and % signal change (average of the significant cluster) in the right middle temporal gyrus, central operculum, and middle frontal gyrus (represented in yellow on brain image) in response to receipt of milkshake taste vs tasteless receipt. Brain images are in radiological format, where the left side of the brain is displayed on the right. All clusters are layered on to the MNI Standard brain for display purposes.

There was greater response to milkshake > tasteless taste in overweight children vs. healthy weight in the right insula/operculum, precentral gyrus, angular gyrus, and middle temporal gyrus, and bilateral precuneus/posterior cingulate cortex (cluster size = 3644 voxels, *p* < .001, *Z* max = 2.84). There were no main effects of CEBQ subscales for taste, but there were interactions between weight status and CEBQ scores (Fig 2). Desire to Drink was negatively correlated with brain response to taste for healthy weight, but positively correlated for overweight in the right postcentral gyrus, parietal operculum, supramarginal gyrus, middle frontal gyrus, and frontal pole (cluster size = 2480 voxels, *p* = .006, *Z* max = 2.78). Opposite patterns were found for Enjoyment of Food, Food Responsiveness, and Emotional Over-Eating, such that positive correlations were found between the scores and brain response to milkshake taste > tasteless taste in healthy weight, but negative correlations found for overweight. For Food Responsiveness, this finding was present in the left putamen, frontal operculum, and postcentral gyrus, and bilateral caudate and thalamus (cluster size = 3347 voxels, *p* < .001, *Z* max = 2.67). For Enjoyment of Food, the finding was present in the bilateral occipital cortex, posterior cingulate, precuneus, angular gyrus, and right middle temporal gyrus (cluster size = 7963 voxels, *p* < .001, *Z* max = 2.76). For Emotional Over-Eating, the finding was present in the right middle temporal gyrus, central operculum, and middle frontal gyrus (cluster size = 2922 voxels, *p* < .003, *Z* max = 2.62).

## Discussion

This study is the first to examine brain response to taste in children younger than 8 years old. It highlights the challenges of this research in young children, as a total of 13 children were excluded due to excessive head motion during scans, including all 4- and 5-year-old participants. Although the sample is small, it provides preliminary and exploratory evidence that brain response to taste in children prior to the onset of obesity may differ from that seen in adult obesity (i.e., heightened response to taste rather than diminished), suggesting the importance of investigating developmental shifts over the course of weight gain. Although preliminary, the lack of differential brain response between groups to the visual cues may suggest an early developmental stage, wherein the cues have not yet taken on the value of the taste reward itself or anticipatory features of reward are not yet developed. Given the small sample and high rate of exclusion of participants and lenient statistical threshold utilized, however, these findings need to be replicated. If replicated in a larger sample with more conservative thresholds, this supports the theory that a greater response to taste may infer risk for developing obesity (due to the presence of heightened response to taste in the overweight group) and over time, neural response may shift to the cue and away from the taste. This shift would result in greater anticipated reward, driving future unhealthy eating and weight gain. Prior studies of older children, age 9–18 years, showed significant differences in brain response to food images between obese and healthy weight children, so the shift may have occurred over that age range, but not for the younger ages of the present study [5,6]. Future longitudinal research is necessary to evaluate this potential developmental shift.

These findings are in line with the prior study of taste response in 8–12 year-old children with greater response to palatable taste in the insula, cingulate, and precuneus/cuneus among obese children [7]. Neither the present study, nor the prior study found significant differences between groups in the striatum, as is commonly seen in adults, which could suggest a developmental shift in the response of that region. Alternatively, the lack of significant difference in striatal response could be due to the small sample and low power to detect the difference.

This study also provides preliminary evidence of the relation between parent-reported child eating behavior and brain response to visual cues and tastes. Because individuals who were overweight with high scores on the Enjoyment of Food, Food Responsiveness, and Emotional Over-Eating scales had lower response to milkshake taste (similar to findings of reduced brain response seen in adults with obesity), perhaps this combination of overweight and eating behavior represents a heightened risk or more advanced brain development toward obesity. The opposite pattern of findings for Desire to Drink, with less response to milkshake taste in healthy weight children with high scores on Desire to Drink, but more response in overweight children, could reflect the relative reward value of milkshake vs. tasteless solution. If overweight children more frequently drink caloric beverages, milkshake could be more reinforcing to those with a high Desire to Drink. Likewise, if healthy weight children with a Desire to Drink are more likely to drink water or low-calorie beverages, milkshake may be less reinforcing.

Overall, this pilot study provides preliminary support for a developmental theory of taste reward for children at early stages of unhealthy weight gain. Overweight children showed a greater brain response to the taste of a milkshake than healthy weight children, although those with higher scores on Enjoyment of Food, Food Responsiveness, and Emotional Over-Eating showed more diminished response, in line with findings of obese adolescents and adults. Further, the study provides evidence of feasibility of brain research on taste in young children. This challenging research is important in targeting at-risk youth to prevent obesity. The number of children who had to be excluded due to motion suggests the need for specific changes to paradigms and apparatus to help children hold still. Changes to the experiment, such as the creation of a more engaging and active taste delivery paradigm, rather than a passive design, may improve children’s ability to remain still. Additionally, modifications to taste delivery manifolds incorporating customized fit and integration with a bite bar system may improve comfort and reduce head motion. Future research is needed to confirm these preliminary findings in larger samples, as well as follow children longitudinally to examine changes in brain response with the development of obesity.
